# Towards Machine Recognition of Facial Expressions of Pain in Horses

**DOI:** 10.3390/ani11061643

**Published:** 2021-06-01

**Authors:** Pia Haubro Andersen, Sofia Broomé, Maheen Rashid, Johan Lundblad, Katrina Ask, Zhenghong Li, Elin Hernlund, Marie Rhodin, Hedvig Kjellström

**Affiliations:** 1 Department of Anatomy, Physiology and Biochemistry, Swedish University of Agricultural Sciences, SE 75007 Uppsala, Sweden; johan.lundblad@slu.se (J.L.); katrina.ask@slu.se (K.A.); elin.hernlund@slu.se (E.H.); marie.rhodin@slu.se (M.R.); 2 Division of Robotics, Perception and Learning, KTH Royal Institute of Technology, SE 100044 Stockholm, Sweden; sbroome@kth.se (S.B.); zhenghong.li@stonybrook.edu (Z.L.); 3 Department of Computer Science, University of California at Davis, California, CA 95616, USA; mhnrashid@gmail.com; 4 Department of Computer Science, Stony Brook University, New York, NY 11794, USA

**Keywords:** pain, facial expressions, objective methods, horse, computer vision, machine learning, deep recurrent two-stream network, convolutional networks, facial keypoint detection, facial action units

## Abstract

**Simple Summary:**

Facial activity can convey valid information about the experience of pain in a horse. However, scoring of pain in horses based on facial activity is still in its infancy and accurate scoring can only be performed by trained assessors. Pain in humans can now be recognized reliably from video footage of faces, using computer vision and machine learning. We examine the hurdles in applying these technologies to horses and suggest two general approaches to automatic horse pain recognition. The first approach involves automatically detecting objectively defined facial expression aspects that do not involve any human judgment of what the expression “means”. Automated classification of pain expressions can then be done according to a rule-based system since the facial expression aspects are defined with this information in mind. The other involves training very flexible machine learning methods with raw videos of horses with known true pain status. The upside of this approach is that the system has access to all the information in the video without engineered intermediate methods that have filtered out most of the variation. However, a large challenge is that large datasets with reliable pain annotation are required. We have obtained promising results from both approaches.

**Abstract:**

Automated recognition of human facial expressions of pain and emotions is to a certain degree a solved problem, using approaches based on computer vision and machine learning. However, the application of such methods to horses has proven difficult. Major barriers are the lack of sufficiently large, annotated databases for horses and difficulties in obtaining correct classifications of pain because horses are non-verbal. This review describes our work to overcome these barriers, using two different approaches. One involves the use of a manual, but relatively objective, classification system for facial activity (Facial Action Coding System), where data are analyzed for pain expressions after coding using machine learning principles. We have devised tools that can aid manual labeling by identifying the faces and facial keypoints of horses. This approach provides promising results in the automated recognition of facial action units from images. The second approach, recurrent neural network end-to-end learning, requires less extraction of features and representations from the video but instead depends on large volumes of video data with ground truth. Our preliminary results suggest clearly that dynamics are important for pain recognition and show that combinations of recurrent neural networks can classify experimental pain in a small number of horses better than human raters.

## 1. Background and Aim

Public concern about equine welfare has increased significantly in recent years, following many reports of wastage and breakdowns in equestrian sport [1,2]. Research across equestrian disciplines has demonstrated that repetitive use injury is the likely precursor to these events [3], so early diagnosis is important. The issue of welfare is pertinent for all stakeholders, from horse owners to horse professionals and veterinarians.

Despite its importance, there is little consensus among veterinarians and laypersons on the quantitative and qualitative aspects of pain in horses. Clinicians often disagree on the intensity of pain in clinical cases, on whether an affective state of a horse is due to pain. As an example of this lack of consensus, practicing veterinarians can score assumed pain in horses associated with a particular condition on a range from “non-painful” to “very painful” [4,5]. For standard surgeries, such as castration, this variation is unlikely to be attributable solely to variations in the display of pain, but rather to lack of consensus regarding pain recognition. Pain is without a doubt developed as a survival parameter [6], and some veterinarians still believe that “suffering promotes survival”—pain is “good” because it serves a protective function. In a Finnish questionnaire study from 2003, 31% of the veterinarians answered that they somewhat agree that a certain level of pain is useful as it prevents excessive movement after surgery while 86% agreed that animals benefit from pain alleviation [7]. A number of contextual factors can influence both the recognition of pain and pain estimates. No horse studies exist, but in dogs and cattle, veterinarians rating of animal pain is influenced by a number of contextual factors including attitudes to animal pain, gender, age and empathy [7,8,9,10,11] Both pain recognition and pain intensity estimation is reduced in human health care providers after repeated exposure to pain in others [12]. This may be a relevant issue for veterinarians witnessing severe animal pain and suffering, as for example lameness in cows, where veterinarians generally scored pain lower than the farmers [13].

The lack of consensus is troubling since veterinary decision-making regarding pain recognition is critical for the care of animals in terms of prescribing pain-alleviating treatments and in animal welfare assessments. Freedom from pain, injury and disease is directly specified as one of the five “freedoms” that constitute the minimal standards of animal welfare in European legislation [14]. Veterinarians are subject to a Code of Conduct drawn up by the licensing authority in their country stating that veterinarians should attempt to relieve animal’s pain and suffering as described for example in the European Veterinary Code of Conduct [15]. Animal pain assessment tools that are objective and feasible are therefore wanted for many reasons.

Some structured tools for pain assessment in horses have been developed in recent decades, mostly for pain diagnosis in specific clinical situations [16,17,18,19]. A pain scale is a formal, structured approach to the assessment of pain. Modern horse pain scales are multi-item scales, based on behavioral and physiological parameters, with the behavioral parameters shown to be more specific to pain than physiological measures [16,17,20,21,22,23]. Physiological parameters, such as heart rate and cortisol blood concentration are correlated significantly with the presence of pain in some studies, but not in others [23]. Physiological parameters may be valid as pain indicators in very controlled settings, but most of them are invasive and require stressful blood sampling or restraint of the animal. Scales comprising the non-invasive observation of body posture, head carriage, location in box and pain behavior, including facial expressions of pain, have been shown to recognize pain in hospital settings [21,24,25], and are therefore interesting targets for automated recognition of pain.

Human research over the past 20 years has shown consistently that facial expressions can be used as tools for recognizing pain in non-verbal humans [26]. Humans seem to be highly specialized for processing facial cues to recognize emotions, including pain, in con-specifics [27,28]. This has proven useful as a tool in pain assessment in non-verbal humans such as infants [29]. Even facial expressions of durations less than 0.5 s may be interpreted [30]. Social ungulates, such as sheep and horses, also use facial visual cues for recognition of identity and emotional state of conspecifics [31]. How humans interpret animal facial cues and vice versa is less researched but interesting from the perspective of the possible automation of the facial expression. No studies have been performed on horses but have been on other species. In an eye-tracking study, Correia-Caeiro et al. [32] investigated how humans and dogs perceived each other’s facial expressions of emotions. While humans modulated their gaze depending on the area of interest, emotion and species observed, dogs modulated their gaze only on the area of interest. The authors suggested that the differences observed could be driven by automatic brain processes adapted for decoding of faces of conspecifics. For humans to recognize the emotional repertoire in another species, it is therefore necessary to employ learning processes that can overrule these apparently automatic brain processes. While the facial musculature is highly conserved across many non-human species, their meaning, and thus the facial expressions of emotions, including the affective components of pain, may likely be species-specific [32]. In the context of this review, their study underlines the need for objective descriptions of facial activity and interpretations not driven by intuition or expectations.

The Facial Action Coding System (FACS) [33] is the current gold standard for the objective measurement of facial movements. FACS is a manual, systematic method for identifying and recording facial expressions, based entirely on the movement of underlying facial muscles. FACS exhaustively describes all observable facial behavior in terms of the muscular actions that comprise it, using elements called action units (AUs), and visibility codes are used when parts of the face are not visible [33]. Each AU, designated by an arbitrary numerical code, denotes the movements of an underlying, anatomically defined, facial muscle. The muscular basis of the facial movements described in FACS has been verified by intramuscular stimulation experiments in humans [34]. FACS coders rely on video observation of facial muscle movements and changes in facial morphology to determine which AU(s) occur. The criteria for coding are described in an anatomical, precise language, which increases the agreement between raters. The inter-observer agreement is good to excellent for spontaneously generated facial behavior in 90% of the AUs in humans [35]. Because facial musculature is conserved across mammal species, with some exceptions regarding nose/muzzle and ears, FACS comparisons can be made across species without interpretation biases or other labels.

FACS has been adapted to several animal species, initially for primates (chimpanzee [36], rhesus macaque [37]; orangutan [38], barbary macaque [37], wild crested macaque [39], Japanese macaque [40], gibbon [41]) and the domestic species such as dogs [42], cats [43] and horses [44]. The development of these modified FACS systems was informed by extensive anatomical work, either through dissection [44,45] and/or intramuscular stimulation of facial muscles in living individuals [34,46].

The FACS standard has been widely adopted by the human research community owing to the exhaustive nature of FACS descriptions [47,48,49,50] and to the fact that FACS can code all possible movements of the face and not only predetermined expressions. The FACS standard for horses, EquiFACS, was developed in 2015 [44] but has only recently been used for the investigation of affective states such as pain [51] and emotional stress [52] in horses. Manual FACS is not suitable as a clinical tool because it requires frame-by-frame coding of video sequences by a trained and certified FACS reader, and is thus extremely resource-demanding, with coding time requirements at least in the range of 1:100 for the average video, one second of video requiring 100 secs of annotation time.

For animals, including horses, “grimace scales” have been developed to standardize the evaluation of facial expressions during pain assessment. These scales require fewer inputs than the FACS-based systems and focus on certain described movements and appearance of ears, eye, side of the chin, nostrils and muzzle/nose/snout. The scales are intended for clinical purposes and can be scored directly or via images. The grimace scales thus lack the dynamic component, which may be essential to determine whether a “grimace” is activated or not, which makes the scoring of grimace scales difficult under dynamic conditions, see, for example, [53]. Generally, rater agreement is much influenced by the quality of the description of the feature rated, with a fuzzy or broad description containing subjective elements giving greater variability. Many grimace scales have good performance parameters, and labels are simple, but their feasibility has not yet been validated, which is delaying the full utilization of these scales as pain assessment tools [54]. One drawback of simplifying labels and/or observation time is that rare or dynamic signs of pain may not be included. A certain feature that appears variably during pain experience will not perform well in assessment tools and may therefore be omitted, despite its possible value as a marker of pain and for the internal validity of the scale.

The development of the many grimace scales clearly shows the need for fast and simple measures of pain. This is also the case for use of facial expressions during complex interactions between animals and humans, such as studies of facial expressions of the horse when moving or being ridden [55,56]. Inspection and annotation of selected images and videos is essential in this type of research, and the selection of horses and footage may be highly prone to different types of bias regarding which footage to select and expectation bias during the subsequent annotation [57].

An objective tool that could recognize pain or facial expressions reliably, rapidly and inexpensively, would therefore greatly enhance research into pain, validation of scales, quality of surveillance and observation of rapidly changing or subtle facial activities, to mention a few advantages.

Computer vision (CV) is an approach for the intelligent processing of images and video. The vast majority of modern CV methods use machine learning (ML) to learn their functions and mappings from data examples. CV/ML is part of the wider field of artificial intelligence (AI) and has now advanced to the point where automatic recognition of human facial expressions [58,59,60,61] can be used in behavioral research and in clinical settings [62,63,64]. Fully automated systems have been developed for recognition of the neutral state and six basic human emotions (anger, disgust, fear, joy, sadness and surprise) in video streams of human faces. For example, Littlewort et al. [59] achieved 88% accuracy in the classification of faked or genuine pain and were also able to code videos and images with action unit activations and pain intensities in real-time.

The major obstruction to the direct application of successful human methods in similar approaches for assessing horse pain is the poor availability of training data. For humans, there are multiple large datasets with image- and video-level expression and action unit annotations [49,65,66], while there are no large publicly available datasets with similar annotations for horses. Good availability of training data would allow modern end-to-end CV/ML techniques, such as those available for humans, to be developed for horses [49,65,66]. The current lack of training data creates a stronger need for hand-engineered algorithms and human labeling and interaction.

Another important obstacle is the lack of a “gold standard” for pain assessment in animals, which, unlike humans, do not have the ability to self-report. Uncertain or incorrect labeling of pain confuses learning algorithms, ultimately hampering detection of pain. Although modern deep neural network approaches are more robust to labeling noise than conventional learning algorithms [67], algorithms, in general, require vastly more training data if the labeling is inaccurate. The performance of automated systems is therefore heavily influenced by the reliability of the annotations [68].

Against this background, the aim of this scoping review was to describe the impact of biological challenges and the opportunities encountered during development work by our research group on CV/ML automated methods for pain recognition in horses. We briefly describe some of our CV/ML research on the path towards automated recognition of pain in horses. Finally, we indicate some future directions for research.

## 2. Biological Challenges and Opportunities in Pain Assessment

Without entering a discussion of definitions and of how pain is “felt” by animals, the difficulties in the correct classification of pain in horses is a core dilemma, not only for the welfare of horses and the success of veterinary practitioners but also for the development of CV/ML approaches for this task. One concrete example of the latter is that ground truth on pain tends to be reduced to binary labels of whether the horse is in pain or not, even when a range of pain intensities can be obtained. This simplification is necessary to obtain a sufficient number of samples per class, despite data scarcity.

The nature of pain is biologically quite complex to address but controversy about the conscious experience of the emotional component of pain in animals is fading [54,69,70], with mounting evidence of an emotional component of pain in all vertebrates [71]. The lack of a gold standard for evaluating the affective states of pain in non-verbal mammals has led to the exploration of bodily behavior or physiological markers to convey information about internal states [72].

The International Association for the Study of Pain IASP defines human pain as “an unpleasant sensory and emotional experience associated with, or resembling that associated with, actual or potential tissue damage” [73]. Because the basic biology and neural apparatus of horses is similar to that of humans, this has led to the use of this definition also for non-human animals such as horses. A review by Sneddon extended this general definition to include that the animal should learn to avoid the noxious stimulus and demonstrate sustained changes in behavior that have a protective function to reduce further injury and pain [69]. While this is perfectly in line with the current understanding of pain-related behavior [74], these criteria are less helpful in the concrete classification of clinical pain. Further, it is generally accepted that no single physiological or biochemical parameter is pathognomonic for pain in horses [24,25], that animals cannot verbalize their pain and that evolutionary heritage may induce prey animals to hide their pain from conspecifics and potential enemies [26,75]. Equids, being prey animals, display pain behaviors that are less obvious to humans [76,77], especially in the presence of unknown or threatening human observers, such as veterinarians. A recent extension to the prey animal narrative is the finding that discomfort behaviors after surgery are expressed less obviously also when a caretaker communicates with the horse, again leading to under-estimation of discomfort [78].

These circumstances can influence both the pain behaviors and the validity of human classification of pain or no pain and may therefore lead to questions about the validity of footage recorded for subsequent CV analysis. This is particularly important if the classification is intended as a label to guide the training of an ML model.

## 3. Requirements on Video Recordings for Use in Computer Vision

In the following section, we list a number of practical issues we have encountered in our interdisciplinary collaboration. Video recordings of horses in the proximity of, or even communicating with, humans should always be labeled accordingly, if used for CV/ML purposes. Before more details emerge about how the presence of humans influences facial expressions, it seems most advisable to use video segments of pain behavior recorded with minimal external influence. Multicamera settings are ideal, especially if both sides of the face should be coded, for example, in laterality studies, or to avoid invisibility. Some of the most widely used horse pain scales involve social interaction between the observer and the horse, that is, touching, feeding the horse or palpating the sore area [18]. A recent study [79] showed that these types of scales generally perform well, but if the pain is evaluated using one of these scales by direct observation, video recordings for CV/ML purposes should be made immediately before the direct pain scoring. It is also important to test the system in another population of horses, to prevent reliance on spurious correlations. Ideally, each horse should be filmed during different levels of pain, to enable a split between model training and test data according to individual subjects. These preliminary criteria are similar to those recommended for pain scale development in general [54]. Post-recording processing requires blinding and randomization before selecting images or videos for annotation, in order to avoid different types of bias, such as selection bias and expectation bias [57].

The demand for video or image quality in CV, in terms of the level of resolution and light conditions, is surprisingly modest. According to CV studies [80] and our experience, 224 × 224 pixels and 25 fps are sufficient for processing images and video in modern CV systems (typically artificial neural networks).

## 4. Will a Pain Scale Deliver Ground Truth?

To determine whether a pain scale can deliver ground truth, it is necessary to know the performance parameters of the pain scale used for the actual population tested during the actual conditions. Surprisingly, few pain scales are adequately validated in this regard [54,79] since sensitivity and specificity can only be measured against ground truth. In horses, a number of pain assessment scales based on facial expressions have been presented recently. In 2014, two independent research groups published novel investigations of facial expressions of pain in horses [81,82], showing that horses exhibit a range of facial expressions when experiencing episodes of acute pain. In one of these studies [81], pain was induced in otherwise healthy horses using known pain induction models, whereas the horses in the other study [82] were clinical cases of hospitalized horses with post-operative pain resulting from castration. Both studies identified changes in the same areas of the face, corresponding to moveable facial muscles related to the ears, eyes, nostrils, lips and chin. While the horses in the castration study had undergone anesthesia six hours before the scoring, the horses in the experimental study were unmedicated but trained to stand in front of the camera. Interestingly, the features described still corresponded rather well to the more formal EquiFACS ontology described by [44], with minor differences, for example, whether the horses in the castration study displayed orbital tightening more often than the experimental horses, which could be a sign of tiredness or sickness. The horse grimace scale has since been used successfully for other painful conditions, such as laminitis [83]. The Equine Utrecht University Scale for Facial Assessment of Pain (EQUUS-FAP) was developed using a number of facial activities, including ear and eyelid position, nostril size and muscle tone of the head and the lip in combination with head movement and specific gross pain behaviors [17]. EQUUS-FAP has since been used to assess pain in horses with colic and head pain [84].

In animals, a correlation between the intensity of facial expression and pain has been reported in mice [85]. Two currently used face-based scales for horses, the Horse Grimace Scale (HGS) [82] and EQUUS-FAP [17,86], use levels of intensity for each individual facial score. For example, the levels in HGS are expressed as “not present, 0 points”, “moderately present, 1 point” or “obviously present 2 points”, where “obviously present” adds double the weight of “moderately present” to the total pain score. In the case of the ears, the different levels represent three different action units, and therefore inferences about correlations between the intensity of an action unit and pain intensity are not justifiable in terms of FACS, but only in terms of grimaces. The Equine Pain Face described by Gleerup et al. [81] does not include the summing of individual facial features, but an observer determines, based on direct observation or from reviewing video recording, whether a pain face is present or not, a process not free of bias. High scores on a pain scale shown to perform well under relevant conditions can be taken to indicate a high likelihood that the horse is in pain. Unfortunately, very few pain scales define cut-off values between “pain” and “no pain”, which is needed for the high usability of a pain scale. For that reason, it is difficult to determine that a horse is not in pain. In some studies, for example [16,19,20], this has led to the inclusion of a subjective assessment of the global pain, which occurs as a category in addition to the otherwise well-defined categories of horse behaviors. For comparison, other pain assessment tools may be added [54]. Subjective assessments, including those provided by expert raters, may be of limited value as ground truth (see e.g., [87]). However, to avoid the logical fallacy of a circular argument, it is of importance to include pain assessments that are not relying on the same categories as investigated in a CV/ML study. If facial action units are to be detected, the pain assessment should then rely on, for example, bodily behaviors. 

Thorough training of the pain rater is important for the reliability of a pain scale. A recent study found that raters of the Horse Grimace Scale showed surprisingly low inter-rater agreement, with a 30-min training session being insufficient for inexperienced raters to obtain satisfactory inter-rater agreement [88]. In a pilot study investigating whether 25 individuals from different backgrounds could assess clinical pain in 18 videos of horses following a 20-min training session on facial expressions of pain, Gleerup et al. [54] found that the participants scored the horses correctly in 61–94% (mean 82%) of the cases. However, the median pairwise Cohen’s Kappa value was 0.48 and the pairwise Spearman correlation of the intensity of the pain face was 0.51, which indicates only modest inter-rater agreement. Movement, stress, coat color and nervous behavior of the horse hampered correct interpretation [89]. Sensitivity and specificity could not be calculated, due to the pilot nature of the study and lack of knowledge of the true pain status of the horses.

In contrast to experimental individuals, clinical cases are often very diverse in respect to age, gender, breed and coat color, all of which can influence pain assessment [90,91]. They are also diverse in terms of temperament [92], earlier experiences and learnings about pain, hospitals, emotional states, transportation and other pain-influencing factors [93,94]. A clinical approach for convergence towards “ground truth” is to record the presence of the (rather few) behaviors reported to be specifically associated with pain, for example, lameness. However, it is debatable whether the intensity of pain is correlated with the degree of lameness if the pain diminishes during unloading of the limb. Objective measurements of perceived sound horses have revealed that 73% show movement asymmetries which might qualify the horse for a full veterinary lameness examination, if referred [95]. It is therefore important to note that not all movement resembling mild lameness is associated with pain, even when measured objectively. In some rare instances, animal experiments may be considered in order to obtain reliable pain labels in cases where clinical data alone cannot provide the information necessary to inform a network. This carries ethical concerns, strict respect for the animal and ethical control. Many management and treatment procedures are indeed quite painful in humans as in horses, and filming of clinical procedures may yield information about facial expressions, which, however, may be blended with other affects. Fully reversible short-term pain induction treatments in horses include a sole pressure model [96], an inflammatory joint model [79,97,98], a non-invasive ischemic pain model [81] and a capsaicin skin sensitization model [81]. An experimental setup allows recording of proper baseline behaviors, while the short-term pain model predicts the time points for pain and subsequent relief of pain. The equine repertoire of facial activities during pain has been shown to be relatively similar for clinical pain [82] and experimental pain [81]. When using experimental pain for the determination of facial activities, validation of the results in clinical pain patients is important [51]. In summary, pain will remain a subjective experience, and there will probably never be a general “gold standard” or biomarker for pain in horses or other animals for that sake. Computer vision and ML methods, therefore, need to circumvent this.

## 5. Analysis of EquiFACS Data

An alternative to the human interpretation of grimaces for assessing pain is the systematic, objective scoring of the visible movement of individual facial muscles over time. This allows the facial repertoire to be fully described and not limited by the categories of the pain assessment tool at stake. The resulting dataset can then be analyzed by data-driven methods for pain or other interpretation after the coding. This means that FACS is not concerned with any theory and the coder need not be familiar with horses or their behavior, which may be an advantage for the blinding procedures which should always be performed. Learning EquiFACS coding is systematized, and learners have to pass a certification exam [44]. In contrast to this, methodologies for analyzing the final FACS dataset are sparse for horses. For humans, Kunz et al. [66] describe the current approaches for the identification of AUs associated with pain. A common method is to apply two criteria: the AU must comprise more than 5% of total pain AU occurrences for coding at a certain frequency and the AU must occur more frequently during pain than during baseline [99]. This method, which is based on an empirical cut-off value of 5%, seems to work well also in horses [51], as it defines AUs and action descriptors (ADs) (facial movements where the muscular basis either cannot be identified or is the result of a different muscle set, e.g., deep muscles). The final ratings are generally in agreement with those obtained using HGS and the pain face category in the Equine Pain Scale [81,82]. However, the method does not take into consideration the temporal aspects of the onset and offset of the various action units. The method also does not define AUs or ADs that might be rare, but important, for pain detection in the horse. We, therefore, developed graph-based statistical methods that describe the *co-occurrence* of AUs and methods for detecting AUs that co-occur (conjoined AUs) over varying periods of time [51,52]. A more complex picture emerged when this co-occurrence method was applied. Chewing (AD81) was found to be important, despite low frequency. *Eye white increase* (AD1) and *inner brow raiser* (AU101) were selected across all observation time lengths. When we used the co-occurrence graph to determine the conjoined pain AUs, we saw that more AUs of the lower face were identified as indicative of pain, including the *chin raiser* (AU17), *nostril dilator* (AD38) and *chewing action* (AD81) identified previously and also the *lip pucker* (AU18) and *upper lip raiser* (AU10). On applying the same statistical methods to sound horses subjected to stressful interventions [52], we observed increased frequencies of *eye white increase* (AD1), *nostril dilator* (AD38), *upper eyelid raiser* (AU5), *inner brow raiser* (AU101) and *tongue show* (AD19), along with an increase in “ear flicker” and “blink frequency”. These results show that ML can be successfully applied on FACS data for horses to reveal more distinct interpretations of the affective states of pain and stress. A limitation of these two very small datasets is that there seems to be some overlap between the facial activities of pain and the facial activities of stress, affecting, for example, the specificity of the findings related to the eye and nostril. This is not surprising, since pain is regarded as an internal stressor and can activate the hypothalamo-pituitary-adrenal axis [100], but it may impair the specificity of face-based pain scales, since high levels of stress may be present during pain evaluations. Furthermore, affective states such as fatigue or residual effects from pharmacological sedatives or anesthetics in the clinical setting may affect how the horse displays pain [101].

Interpretation of the dynamics of facial expressions is an important road forward. Wathan et al. [44] claim that certain facial movements can only be distinguished accurately from sequences. Our FACS-based results seem to corroborate this, an example is the identification of increased frequency of the *half blink* (AU47) as a new indicator for horses in pain in [51], but further research is needed on interpretation of facial dynamics during mixed affective states. The importance of the loss of temporal information in still images of humans is discussed by Kunz et al. [102], who showed that not all core features of a pain face are present at the same time in all individuals. The frequencies of occurrence of the prototypical pain expressions ranged from 10% to 60%, leading the authors to conclude that the likelihood that all four key facial activities occurred simultaneously might be very low. Similarly, we found that only a very small proportion (6.1%) of frames in the pain videos contained three or more pain AUs [51]. This impedes accurate pain assessment on the basis of randomly selected frames, as the chances of accurately assessing a frame as a horse in pain would be only 6.1%, making this method very insensitive for recognition of pain. Longer observation times are therefore necessary. Automated detection of facial activities may solve some of these issues relating to large differences between the scoring of frames versus direct scoring from video, as already addressed by [53].

## 6. Automated Extraction of Facial Features from Images

Automated pain detection based on EquiFACS in horses requires preliminary efforts to detect and locate a horse face in an image or video clip and to detect individual (EquiFACS) action units. Existing standard methods within CV/ML for object detection can be fine-tuned to recognize specific object classes. In the “Horse Face Finder” [103] we fine-tuned an object detection neural network to detect frames when a horse shows its face to the camera, which further distinguished between different angles of the face (side-view or a 45-degree view relative to the camera), from videos of horses standing in a box. This is an important aid for the otherwise time-consuming selection of sequences from videos that are usable for annotation of equine facial expressions. Importantly, this tool can help reduce selection bias when studying facial expressions in horses using video recordings.

Importantly, the Horse Face Finder enables facial expression analysis of videos of unrestrained horses in arbitrary positions relative to the camera. As a result, human supervision of the horse before or during filming becomes unnecessary. In fact, human expression datasets such as [49,65,104] that show human faces in full frontal view of the camera are not only difficult to collect but have limited generalization to natural settings where a face is likely to move in and out of the camera view. As a result, face detection and alignment—via facial keypoint detection—are standard preprocessing steps to expression analysis, for example as in [105].

### 6.1. Animal Facial Keypoint Detection

Facial keypoints are points in an image or video that indicate the location of particular anatomical regions of the face. They are important for registering the facial image and for extracting useful features around facial parts that visually change with AU activation. Training a convolutional neural network (CNN) from scratch typically requires a large amount of labeled data, which can be time-consuming and expensive to collect. In contrast to human datasets with keypoint annotations (e.g., 26,000 images in [106]), there are no large datasets of animal facial keypoints (e.g., the sheep dataset from [107] has only 600 images). Transfer learning from larger to smaller datasets is widely used within deep learning [108], but the structural differences between human and animal faces prohibit direct fine-tuning (i.e., continued training) of a human keypoint detector with animal training data.

**Figure 1:**
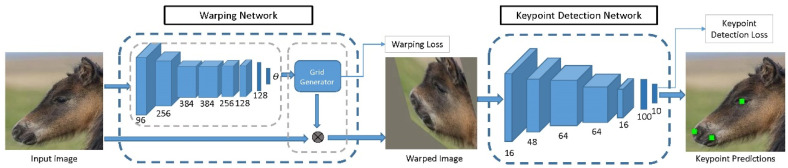
A deep convolutional neural network (CNN) for animal facial keypoint detection. It produces keypoint predictions for the left eye, right eye, nose, left mouth corner and right mouth corner. From [109].

For this reason, we developed a method for the key purpose of adapting animal training data to the pre-trained human keypoint detector, so that it is better conditioned for fine-tuning [109]. Specifically, we first brought the shapes of animal and human faces closer together by morphing animal faces to resemble the human shape. We then used the resulting warped images to fine-tune a deep network trained on human facial keypoint data to detect animal facial keypoints. In this way, we were able to transfer information between human and animal keypoint data, which resulted in more accurate keypoint detection on animal faces, while reducing the required amount of annotated data. Figure 1 shows an overview of the method.

Using the method, we achieved state-of-the-art performance in both horse and sheep facial keypoint detection, with 8.36% and 0.87% failure rates, respectively. The large dataset of 3717 images of horse faces with horse facial keypoint annotation, collected by keyword searches on public search engines and manually annotated for keypoints is publicly available in [109].

### 6.2. Automated Detection of Facial Action Units

With the promising results from the manually annotated EquiFACS datasets [51,52], we investigated methods for automated recognition of horse facial AUs in still images [110]. Previous work has explored automated detection of keypoint-based facial expression information, but in a simplified form compared to, for example, EquiFACS [111,112,113]. In studies by the authors of [111,112], the method learned to classify the appearance of certain facial areas directly in terms of pain, meaning the presence of a specific AU, was not determined. In Lu et al. [111], the system was trained to recognize and score the intensity of nine different facial areas on a scale between 0 and 2. These three approaches were developed for sheep, horses and donkeys and all rely on handcrafted feature representation of image patches (Histograms of Oriented Gradients), although [112] includes some experiments on CNN features. This is a robust and classical method, but it limits the range and precision of expressions that can be represented.

Despite the very different facial configurations of horses and humans, a remarkably high number of AUs are conserved across species [47]. We, therefore, aimed at applying methods for human AU detection to horses (Deep Region and Multi-label Learning, DRML [114]).

We used our entire pool of EquiFACS annotated data, collected for various purposes in our research group. This yielded more than 20,000 labeled short video clips, representing 31 AUs, including ADs and ear EADs. In experiments, we trained separate binary classifiers for the nine AUs with the largest sample sizes in our dataset (according to a pre-set threshold), in order to have enough data for the training to converge properly. Even after the selection of the nine largest classes, significant class imbalance among these remained. For this reason, we resorted to training separate classifiers for each action unit. However, in a future scenario with access to more and varied labeled AU data, it would be interesting to train a fine-grained AU classifier jointly on multiple classes.

With the *inner brow raiser* (AU101) as a model, we evaluated the performance of two common classifiers, DRML and AlexNet, to detect facial action units. Prior to that, we extracted face regions and eye regions using a standard object detection pipeline. We evaluated the performance gain going from raw frames (where the relevant area in the case of facial AUs in surveillance videos of horses is an extremely small part of the frame), to face crops, to eye region and lower-face crops of the horse in the frame, zooming in on the fine-grained muscular action to be detected. For raw frames and face crops, both classifiers failed to focus on relevant anatomical regions. Based on this, we decided that it was critical to provide a pre-defined region-of-interest (ROI) as input to the classifiers, to help focus on the correct anatomical region. Note that for a specific pre-defined ROI, the deep region method in DRML is not suitable. Instead, we kept the main network architecture of the DRML and replaced the deep region layer with a single convolutional layer. 

**Figure 2:**
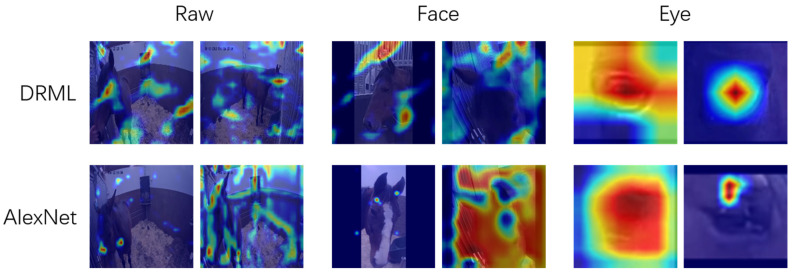
Grad-CAM saliency maps of the models for binary classification of action unit AU101. When raw images were used (left), neither the DRML nor AlexNet model could identify AU101. When the eye region was extracted as a region-of-interest (right), the activity of AU101 was detected by both models but was most recognizable in the AlexNet image, lower row, last image. From [110]. A video clip with an example of AU101 is given in the Supplementary Material.

If the pre-defined related regions were cropped out before training for classification, the classifiers were able to address the correct regions, such as the eyelid, nostril, corner of mouth and tongue in many cases (for the example of AU101, see Figure 2). Our next step will be to compare the performance with hand-labeled films.

The framework, which took single frames as input, did not work for the *ear action* descriptors. This is likely due to the many different positions possible for ears, which should, therefore, be examined in video and not in images. This is probably a large and general problem in equine face-based research. Therefore, a future direction is to extend our EquiFACS detection method to the temporal domain.

## 7. Automated Pain Detection Using Temporal Information

The above-mentioned methods for horse pain detection are based on still images, which means that temporal information is not available, and the frames are treated as independent. It has been shown for both humans [115,116] and horses [81], that temporal information is important when a human interprets signs of pain. We, therefore, developed a second method built on recurrent neural networks (RNNs) receiving videos of horses as input. The input videos showed the head and neck of the horses as they were standing in front of the camera, both during experimentally induced acute pain and in their baseline conditions [117]. These RNNs operate on sequences of images by passing frames through computational nodes, sequentially updating their so-called hidden states, each of which should encapsulate what a node has learned in the process. In our scenario, that is, training the RNN to classify pain, the hidden states of the recurrent nodes in the network should contain information pertaining to pain behavior, or lack thereof. The training procedure is the same as for the ML systems (neural networks) described above in that it is supervised. This means that the system is trained by being shown labeled examples of the different classes and then mathematically prompted to update its parameters in a particular direction when it makes faulty classifications during training.

**Figure 3:**
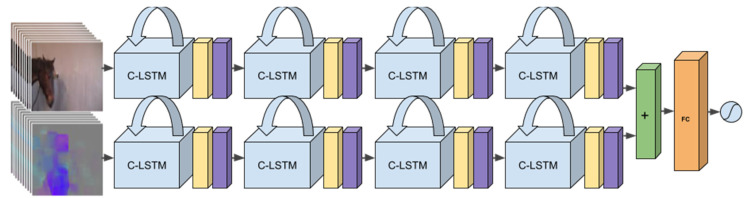
Deep recurrent two-stream network for end-to-end detection of pain from a sequence of Red-Green-Blue (RGB) images and optical flow images. Each optical flow image is computed from two subsequent RGB images using standard image processing techniques. From [117].

Figure 3 shows the neural network architecture, which consists of two different streams, one with the raw RGB (Red-Green-Blue, standard notation for color in images) video frames as input and one with the optical flow (the motions of pixels between two images adjacent in time) as input. Although motion is captured through the sequences of frames (RGB input is given at 2 fps), the optical flow is computed at a higher frame rate and can capture motion that is faster and of shorter duration, that is, more fine-grained motion capture. The optical flow can also serve to highlight where interesting motion is happening in the input sequence, which aids the network during training. Thus, the two streams are complementary. The two streams are processed separately through several layers and fused late in the network structure. The methodology and the reasons for the design choices we made are further explained in [117].

This was in fact the first successful attempt to perform pain recognition in any non-human species by extracting spatiotemporal patterns from video data. Since then, Pessanha et al. [113] have explored the temporal dimension in pain recognition and disease progression monitoring from video data of sheep. Their study was performed on animals in the wild, over a time span of one month, although the image processing was done at frame level and classifications were subsequently averaged over time. Prior to our work, Tuttle et al. [118] had trained a system to recognize pain in mice and observed improved performance by averaging the results from multiple frames over time, but the patterns detected were still found separately, in single images. It is computationally less expensive to perform single-frame analysis that is subsequently aggregated, but there are many fine-grained patterns that cannot be detected in this way (such as distinguishing between the two EquiFACS units blink (AU145) and half blink (AU47), as noted in [110]).

Another important methodological advantage of the RNN approach (illustrated in Figure 3) is that it learns patterns end-to-end, in a completely data-driven fashion, directly from the raw RGB video and without the help of intermediate representations, in contrast to the EquiFACS-based pain detection method described above. More fine-scale variations that might be lost in the intermediate EquiFACS interpretation can be captured. An end-to-end method also has exploratory potential in its possibility for identifying behaviors not previously associated with pain by veterinarians. However, the downside compared to FACS-based methods is that the method requires much more training data, which is an important factor given that data collection with a valid gold standard, that is, ground truth, is cumbersome. The use of EquiFACS as a non-interpretative description of changes in facial expression may therefore be of great value to overcome data scarcity.

The neural network was both trained and tested on mutually exclusive parts (subject-wise) of the dataset described in [117]. Quantitative tests showed that the neural network outperformed a group of human veterinarians assessing pain by watching blinded video clips by 54.5% for humans and 73.5% (macro average F1-score) for the two classes.

**Figure 4:**
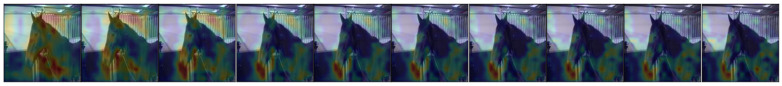
Ten frames from a sequence of a horse not in pain that was misclassified by human experts, correctly classified by the neural network. Overlaid on each video frame is an illustration of where in the video frame the neural network focused, based on color changes. From [117].

A qualitative evaluation was performed using Grad-CAM [119], a method for visualizing areas within an image on which the neural network focuses for a particular classification decision. Figure 4 shows frames from a video of a horse not in pain, as correctly classified by the RNN method, but classified as being in pain by the veterinarians in the blinded study. Red regions correspond to areas in the video frame where Grad-CAM detected a strong focus by the neural network. The frequent attention paid to the muzzle, ears and eyes indicates that these areas carry information about pain, which is in accordance with EquiFACS and grimace scales described above.

## 8. Discussion and Concluding Comments

The aim of this scoping review was to share efforts aimed at automating the process of recognizing and assessing pain, using the horse as a model agricultural species. We chose the horse as a model for several reasons. First, a number of tools for the assessment of acute pain have been developed and used for the horse, providing the possibility to obtain ground truth. Second, ethograms of behavior in healthy and pain-affected horses exist, providing targets for CV techniques. Third, facial expressions of pain and a facial action coding system for horses have been described, providing the basis for use of CV/ML techniques for pattern recognition. Extending machine recognition of pain to any other agricultural species would have a positive welfare impact, because frequent, objective, reliable and inexpensive information can be obtained remotely. The rather optimistic view that the very successful human methodology can be applied to animals is only partly substantiated. We have successfully morphed animal features to human features and then used a human database to improve results in our keypoint detection method. Since facial muscles are well conserved across mammal species, also between horses and humans [44] we used DRML, a standard model for human facial AU detection, and trained it to detect specific facial AUs in certain anatomical regions. The correct classification rates were promising, but more research is needed before automated recognition of AUs can be reliably used in a real-world setting for horses.

We have also performed end-to-end binary pain recognition in videos of horses using a two-stream recurrent neural network. This method can outperform blinded veterinarian experts in the task and is the first to extract spatiotemporal patterns from sequences of images to discern pain behavior from facial expressions.

There is a lack of databases containing annotated horse videos together with adequate metadata, such as the results of observer-based pain scoring or experimental pain status. We have published videos and EquiFACS annotation for six horses [51], but further research on such a small dataset carries a high risk of bias. From an ethical perspective, horse pain researchers should combine their databases. We have developed a tool for unbiased selection of horse faces in videos [103], which we have made freely available. The tool drastically reduces video observation time, but efforts should be made to combine forces and create a dataset that can be used by many researchers. From an ethical point of view, all studies that involve experimental pain in animals should share data. Although scales may be constructed and used under different conditions and facial activities may be defined differently between pain scales, such databases would represent the diversity of pain and other affective states, such as fear and stress. 

Kunz et al. [102] showed that there is more than one prototypic pain face in humans, and in many instances, there is also an overlay of non-pain-related AUs. It is possible that many different “faces of pain” exist also in horses, and the FACS methodology we propose in this review may contribute to a much more diverse and maybe also more complex truth about pain expressions, than what appears in our current equine facial expressions pain scales. Six different horse personalities, or “horsonalities”, have been identified by Lloyd et al. [120], and these may indirectly influence the display of pain behavior. Also, the influence of type and duration of pain need to be researched, since there are hypotheses on the differences in facial expressions of pain between acute nociceptive pain and chronic pain already brought forward by for example [26,76,121,122] which could be tested.

For the biological aspect of pain, the next step must be to provide more EquiFACS-based knowledge on the display of pain—and no-pain—under conditions involving external input, such as observer presence, movement or under a rider. This should be combined with continued efforts towards automated detection of AUs, to prolong observation times. Automated recognition of facial AUs and/or pain and stress would greatly enhance research possibilities to prolong the windows where pain and other affective states can be observed. This would provide new knowledge about pain, and perhaps also other affective states of the horse. Because of the adaptation of the FACS for many other mammal species, the research described here can be adapted to other research areas and eventually provide information that can revolutionize the way we look at animals.

## Supplementary Materials

The following are available online at https://www.mdpi.com/article/10.3390/ani11061643/s1, Video S1 (file AU101_horse 9). The 4 s video clip shows the eye region of a horse displaying *inner brow raiser* (AU101) by contraction of the levator anguli oculi medialis muscle, resulting in bulging skin in the inner corner of the eye, deepened wrinkles and the angular shape of the eyelid. The video is best watched in slow forward or frame by frame. The horse is from the study in [79].
